# Responses of *Anabaena* sp. PCC7120 to lindane: Physiological effects and differential expression of potential *lin* genes

**DOI:** 10.1002/mbo3.1355

**Published:** 2023-04-27

**Authors:** Jorge Guío, Maria F. Fillat, Maria L. Peleato, Emma Sevilla

**Affiliations:** ^1^ Departamento de Bioquímica y Biología Molecular y Celular and Institute for Biocomputation and Physics of Complex Systems Universidad de Zaragoza Zaragoza Spain

**Keywords:** aquatic bacteria, biodegradation, cyanobacteria, environmental microbiology

## Abstract

Lindane (γ‐HCH) is an organochlorine pesticide that causes huge environmental concerns worldwide due to its recalcitrance and toxicity. The use of the cyanobacterium *Anabaena* sp. PCC 7120 in aquatic lindane bioremediation has been suggested but information relative to this process is scarce. In the present work, data relative to the growth, pigment composition, photosynthetic/respiration rate, and oxidative stress response of *Anabaena* sp. PCC 7120 in the presence of lindane at its solubility limit in water are shown. In addition, lindane degradation experiments revealed almost a total disappearance of lindane in the supernatants of *Anabaena* sp. PCC 7120 culture after 6 days of incubation. The diminishing in lindane concentration was in concordance with an increase in the levels of trichlorobenzene inside the cells. Furthermore, to identify potential orthologs of the *linA, linB, linC, linD, linE*, and *linR* genes from *Sphingomonas paucimobilis* B90A in *Anabaena* sp. PCC 7120, a whole genome screening was performed allowing the identification of five putative *lin* orthologs (*all1353* and *all0193* putative orthologs of *linB, all3836* putative orthologs of *linC*, and *all0352* and *alr0353* putative orthologs of *linE* and *linR*, respectively) which could be involved in the lindane degradation pathway. Differential expression analysis of these genes in the presence of lindane revealed strong upregulation of one of the potential *lin* genes of *Anabaena* sp. PCC 7120.

## Graphical abstract

In this article, the tolerance of *Anabaena* sp. to lindane was studied as well as the expression of its potential *lin* genes. Our goal was to obtain information to create strategies for lindane bioremediation and also to find the induction of genes in the presence of lindane that can be used in the development of whole‐cell biosensors.

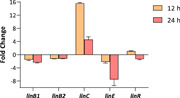

## 1 INTRODUCTION

Hexachlorocyclohexane (HCH) was widely used as a pesticide between 1930 and 1990, being first applied as a technical mixture that contained α‐HCH, β‐HCH, γ‐HCH, δ‐HCH, and ε‐HCH. HCH is usually synthesized by benzene photochlorination, a process in which the aforementioned isomers were formed. Afterward, solely lindane (γ‐HCH isomer) was commercialized because, among all the isomers, only γ‐HCH had strong insecticidal properties (Li, 1999). Since the industrial procedure rends only about 10%–15% of γ‐HCH, it has been estimated that, in those years, between four and six million tons of the other HCH isomers were dumped worldwide generating an important environmental and health concern (Lal et al., 2010). In 2009, HCH isomers were defined as persistent organic pollutants in the Stockholm Convention (2009) and since 2015 lindane was included in the list of carcinogenic agents for humans by the World Health Organization (2018). Although HCH is durable and recalcitrant, some microorganisms are capable of degrading one or more HCH isomers (Lal et al., 2010). The best characterized in terms of their lindane biodegradation abilities are heterotrophic bacteria belonging to the Sphingomonadaceae family (Verma et al., 2014). The diversity, organization, and distribution of lindane catabolic pathways are reasonably well‐established in these bacteria (Verma et al., 2014). Aerobic lindane degradation pathways are conformed by *lin* genes and are subdivided into upper and lower pathways. In particular, the *lin* pathways are well‐characterized in *Sphingobium japonicum* UT26S (Nagata et al., 1999, 2007) and *Sphingobium indicum* B90A (Lal et al., 2010). The upper pathway comprises *linA* (HCH dechlorinase), *linB* (haloalkane dehalogenase), and *linC* (dehydrogenase) whereas the lower pathway is formed by *linD* (reductive dechlorinase), *linE* (ring cleavage oxygenase), *linF* (maleylacetate reductase), *linG,H* (acyl‐CoA transferase), *linJ* (thiolase) and *linI* and *linR* (transcriptional regulators) (Nagata et al., 2007; Verma et al., 2014). The dynamics of *lin* genes expression in *Sphingomonas paucimobilis* B90A (also known as *S. indicum* B90A) revealed that *linA*, *linB*, and *linC* were expressed constitutively whereas, on the contrary, the expression of *linD* and *linE* was induced in presence of 7 mg/L of lindane and 2 mg/L of α‐HCH (Suar et al., 2004).

Among photoautotrophic organisms, *Anabaena* sp. PCC 7120, *Nostoc ellipsosporum*, and *Microcystis aeruginosa* PCC 7806 have been described as potential lindane degraders (Ceballos‐Laita et al., 2015; Kuritz & Wolk, 1995; Kuritz et al., 1997; Sarasa‐Buisán et al., 2022). *Anabaena* sp. PCC 7120, a nitrogen‐fixing cyanobacterium, was reported to be able to metabolize lindane (γ‐HCH) yielding pentachlorocyclohexene and 1,2,4‐trichlorobenzene in a process dependent on *nir* operon, which encodes some enzymes for nitrate utilization (Kuritz & Wolk, 1995; Kuritz et al., 1997). However, the genes and subsequent catabolic pathways involved in such a process remain unknown. In addition, the effect of the presence of lindane on the photosynthetic apparatus of *Anabaena* sp. PCC 7120 was analyzed, concluding that photosynthetic activity was slightly increased in the presence of lindane and that no changes were observed in the synthesis and activity of ferredoxin‐NADP^+^ reductase (FNR) (Bueno et al., 2004).

On the other hand, it was previously reported that the levels of lindane in supernatants of *M. aeruginosa* PCC 7806 cultures treated with 7 mg/L lindane decreased significantly after 15 days of treatment (Ceballos‐Laita et al., 2015). Nevertheless, only an ortholog of the *linC* gene was found in the genome of *M. aeruginosa* NIES‐843, which interestingly was induced in the presence of lindane (Sarasa‐Buisán et al., 2022).

The *lin* pathway probably constitutes a unique biochemical system that allows HCH detoxification. Until the moment the genes that constitute this pathway and the substrate specificity, isomer coverage, and kinetics of enzymes are relatively well‐known in Sphingomonads. However, information concerning this pathway in other organisms is scarce (Lal et al., 2010). The knowledge of the presence of *lin* genes as well as the tolerance of *Anabaena* to lindane is important to evaluate the potential use of this model cyanobacterium in bioremediation strategies or in other processes such as the development of whole‐cell biosensors to detect HCH isomers.

In this work, *Anabaena* sp. PCC 7120 physiological responses to the presence of lindane, namely growth rate, pigment composition, photosynthetic/respiration rates, and oxidative stress response were evaluated. Moreover, a search of *lin* genes in the genome of this cyanobacterium was performed and their transcriptional responses in the presence of lindane were studied. Finally, *Anabaena* sp. PCC 7120 degradative capacity was also studied by testing the disappearance of lindane in the supernatants of cells treated with this pesticide as well as the appearance of lindane metabolic intermediaries inside *Anabaena* sp. PCC 7120 cells.

## 2 MATERIALS AND METHODS

### 2.1 Strains and culture conditions

*Anabaena* sp. PCC 7120 was grown photoautotrophically in BG‐11 medium (Stanier et al., 1979) containing 30 μM of FeSO_4_ instead of 30 μM of ammonium ferric citrate to avoid inhibition of lindane metabolization (Kuritz & Wolk, 1995). These cultures were maintained in 250 mL Erlenmeyer flasks holding 100 ml of *Anabaena* sp. PCC 7120 cultures each at 28°C in a New Brunswick™ Innova® 43 Shaker under continuous illumination with white light at 30 μmol photons m^−2^ s^−1^ and gentle shaking at 100 rpm. To study the effects of lindane (γ‐HCH) on *Anabaena* sp. PCC 7120, 10 μL of a solution of 70 mg/mL of γ‐HCH in DMSO were added to 250 mL Erlenmeyer flasks containing 100 mL of culture, obtaining a final concentration of 7 mg/L. The effect of DMSO on *Anabaena* sp. PCC 7120 was also analyzed by adding 10 μL of DMSO to 250 mL Erlenmeyer flasks containing 100 mL of culture.

### 2.2 Growth measurement

The effect of 7 mg/L of γ‐HCH on the photoautotrophic growth of three independent cultures of *Anabaena* sp. PCC 7120 with an initial OD_750nm_ of 0.3 was measured spectrophotometrically using a Cary 100 Bio UV‐Visible spectrophotometer (Varian). The optical density was recorded at 750 nm for 22 days every 48 h. At the same time, the packed cell volume (PCV) was measured using 5 mL graduated tubes with a capacity of 60 μL of packed volume. Five milliliters of cultures were centrifuged in the aforementioned tubes for 5 min at 18°C and 2000x*g* using an Allegra X 30 R centrifuge. PCV measurements were expressed as microliters of PCV per milliliter of culture.

### 2.3 Pigment content determinations

Pigment content of *Anabaena* sp. PCC 7120 in the presence of 7 mg/L of γ‐HCH was determined at exponential (OD_750nm_ = 1.0) and stationary (OD_750nm_ = 2.0) phases of growth in three independent cultures of each condition with an initial OD_750nm_ of 0.3. Chl *a*, phycobiliprotein, and carotenoid levels were quantified as described by Mackinney (1941), Glazer (1976), and Davies (1976), respectively.

### 2.4 Net photosynthesis and dark respiration rate measurements

Net photosynthesis and dark respiration rates of *Anabaena* sp. PCC 7120 were measured at room temperature with a Clark‐type oxygen electrode model Chlorolab 2 (Hansatech). Net photosynthesis (defined as true photosynthesis minus photorespiration and dark respiration (Wohlfahrt & Gu, 2015), was determined by measuring the O_2_ increase during 5 min illuminating cell suspensions with white light at 10, 50, 100, and 400 µmol photons·m^−2^·s^−1^. Dark respiration was determined by measuring the consumption of oxygen during 3 min in darkness. Both rates were measured at exponential (OD_750nm_ = 1.0) and stationary (OD_750nm_ = 2.0) phases of growth and were expressed as pmol O_2_ × s^−1^/μL of PCV. All measurements were performed in triplicate.

### 2.5 Catalase and superoxide dismutase (SOD) activity determinations

Catalase and SOD activities were measured after 48 h of exposure to 7 mg/L of lindane in three independent cultures with an initial OD_750nm_ of 0.3. 50 mL of cell culture were centrifuged at 3000 g and 4°C for 10 min and cells were resuspended in 1 mL of phosphate buffer (KH_2_PO_4_/K_2_HPO_4_) 50 mM pH 7. Then cells were broken by sonication, the resulting solution was centrifuged at 13,000 g and 4°C for 10 min and the total protein concentration was determined in the supernatant by using the BCA™ Protein Assay kit (Thermo Fisher Scientific).

Catalase activity was determined as described by Beers and Sizer (1952). Six hundred micrograms of protein extract was mixed with H_2_O_2_ to a final concentration of 20 mM and the dissociation of H_2_O_2_ was followed at 240 nm with a Cary 100 Bio UV‐Visible spectrophotometer (Varian) for 5 min. Catalase activity was expressed in units per milligram of total protein, defining a unit as the amount of enzyme that dissociates 1 μg of H_2_O_2_ per minute.

SOD activity was measured as described by Winterbourn et al. (1975) with some modifications implemented by Sein‐Echaluce et al. (2015). Six hundred micrograms of protein extract was mixed with 6.4 mM ethylenediaminetetraacetic acid (EDTA), 41 μM nitro‐blue tetrazolium (NBT), 2.3 μM riboflavin and 23.5 μM N,N,N′,N′‐Tetramethylethylenediamine (TEMED) and SOD activity was determined by measuring the inhibition of NBT reduction by SOD. Thus, absorbance at 560 nm was determined before and after illuminating the mixtures for 10 min with UV light, using a control containing phosphate buffer instead of the protein extract. SOD activity was expressed in units per milligram of total protein, defining a unit as the amount of enzyme that inhibits the maximum reduction to half.

### 2.6 Microscopy experiments

To analyze the effects of lindane on the morphology of *Anabaena* sp. PCC 7120 filaments, bright‐field, and fluorescence microscopic examinations were carried out with a Nikon Eclipse 50i Epi‐fluorescence microscope. Photographs were taken with a Nikon DXM1200F camera coupled to the microscope both at exponential (OD_750nm_ = 1.0) and stationary (OD_750nm_ = 2.0) phases of growth. Fluorescence was detected using a 560/40 nm excitation filter, a 595 nm dichroic beam splitter, and a 630/60 nm emission filter.

### 2.7 Identification of putative *lin* genes in the genome of *Anabaena* sp. PCC 7120

To identify putative *lin* genes in the genome of *Anabaena* sp. PCC 7120 the sequences of the proteins encoded by genes *linA*, *linB*, *linC*, *linD*, *linE*, and *linR* from *S. paucimobilis* B90A were retrieved from Uniprot (https://www.uniprot.org/). Using these sequences as queries and an expectancy‐value threshold of 0.005, a protein BLAST (Basic Local Alignment Search Tool) using the CyanoBase Similarity Search (http://genome.microbedb.jp/blast/blast_search/cyanobase/genes) was performed on *Anabaena* sp. PCC 7120 genome. In all cases, the sequence with the lowest expected value was selected and a pairwise global alignment using ClustalW (https://www.genome.jp/tools-bin/clustalw) was performed using default settings.

### 2.8 Degradation measurements

The degradation of lindane was studied in three independent cultures with an initial OD_750nm_ of 0.6 containing 7 mg/L of lindane. Erlenmeyer flasks containing 7 mg/L of lindane in BG‐11 medium without cells served as controls for evaporation and photodegradation. After 1, 3, and 6 days of treatment, 10 mL of each sample was taken and centrifuged for 5 min at 3000 g in the case of *Anabaena* sp. PCC 7120 cultures. Lindane analysis of these samples was carried out in the HCH Analysis laboratories at the facilities of the new security cell in Bailín II (Sabiñánigo, Huesca). The isomers of HCH in an aqueous matrix were extracted using a liquid‐liquid extraction with hexane and the quantification of the remaining amount of each isomer was carried out using gas chromatography with a mass detector (Agilent Series 7890 A). The identification of isomers was carried out using commercial individual standards.

The formation of degradation intermediaries was studied in three independent cultures with an initial OD_750nm_ of 0.6 containing 7 mg/L of lindane. Ten milliliters of cultures were taken after 0 (control), 24, and 48 h of exposure to lindane and then centrifuged for 5 min at 3000 g. A pellet of cells was extracted by adding 2 mL of hexane at 4°C and shaking in a vortex for 5 min. Finally, the resulting suspension was centrifuged at 12,000 g and 4°C for 10 min and the supernatant was used to determine the presence of lindane degradation intermediaries. One microliter of diluted samples were analyzed in a gas chromatography coupled to a mass detector using a 436GC Chromatograph equipped with a Rxi‐5Sil MS column (30 m × 0.25 mm × 0.25 μm). Gas chromatography/mass spectrometry (GC‐MS) analyses were performed at the Interdepartmental Research services of the Autonomous University of Madrid, Spain.

### 2.9 RNA extraction and real‐time RT‐PCR

The effects of lindane on gene expression were analyzed in three independent cultures of *Anabaena* sp. PCC 7120 with an initial OD_750nm_ of 0.3. RNA was extracted from 25 mL of each culture after 12 and 24 h of exposure to 7 mg/L of lindane following a method described in Sarasa‐Buisan et al. (2022). The absence of DNA in the RNA samples was checked by real‐time PCR, using oligonucleotides for the housekeeping gene *rnpB* (Vioque, 1992). RNA was quantified spectrophotometrically using a SPECORD® PLUS Analytik Jena spectrophotometer.

Two micrograms of total RNA was reverse‐transcribed using SuperScript retrotranscriptase (Invitrogen) following the manufacturer's conditions. Real‐time PCR was performed using the ViiA™ 7 real‐time PCR System (Applied Biosystems). The specific primers for each gene that were analyzed are included in Appendix: Table A1. Each reaction was set up by mixing 12.5 µL of SYBR Green PCR Master Mix with 0.4 μL of 25 µM primer mixture and 10 ng of cDNA template in a final volume of 30 µL. The extension of PCR products was performed at 60°C. The relative mRNA levels of the target genes were normalized to the housekeeping gene *rnpB* (Vioque, 1992). Relative quantification was performed according to the comparative Ct method (ΔΔCt Method) (Livak & Schmittgen, 2001). The minimum fold‐change threshold was set up to ±1.5‐fold.

### 2.10 Statistical tools

To determine whether the changes observed in the measurements of different parameters were significant, *t*‐test statistical analyses were performed by using the GraphPad Prism 7 program.

## 3 RESULTS

### 3.1 *Anabaena* sp. PCC 7120 cells exhibited a high level of tolerance to lindane

**Figure 1:**
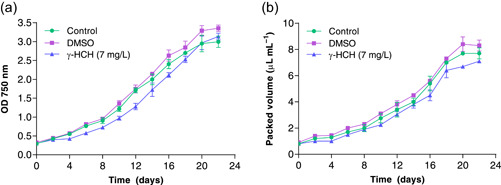
Growth curves of *Anabaena* sp. PCC 7120 in the presence of 7 mg/L of lindane for 22 days. (a) Optical densities at 750 nm of *Anabaena* sp. PCC 7120 cultures treated with 7 mg/L of lindane in comparison to untreated cells. Since DMSO was used to dissolve lindane in the preparation of stock solutions, a control culture in which the same amount of DMSO alone was added to cells is also included. Three biological replicates were used for each sample. The average of the three measurements is represented and the standard deviation is included. (b) Packed cell volume of lindane‐treated cells in comparison to untreated cells. Again, a control culture with DMSO alone was also included. Three biological replicates were used in each sample. The average of the three measurements is represented and the standard deviation is included.

*Anabaena* cells were exposed to 7 mg/L of lindane for 22 days in batch cultures. The concentration added to *Anabaena* cultures corresponded to the solubility limit of lindane in water. The growth rate was measured by using the optical density at 750 nm as well as the PCV (Figure 1a,b). Since DMSO was used to solve lindane, a control culture in which DMSO alone was added to the cells was monitored. Growth curves representing both parameters, OD at 750 nm and PCV showed similar growth of *Anabaena* in the presence and absence of lindane (Figure 1a,b). Indeed, the doubling time was 5.28 ± 0.051 d^−1^ for *Anabaena* sp. PCC 7120 in the absence of lindane and 5.02 ± 0.47 d^−1^ in the presence of lindane. *t*‐test statistical analysis of these data revealed that the change observed in the doubling time in the presence of lindane is not significant (*p*‐value = 0.3948).

**Figure 2:**
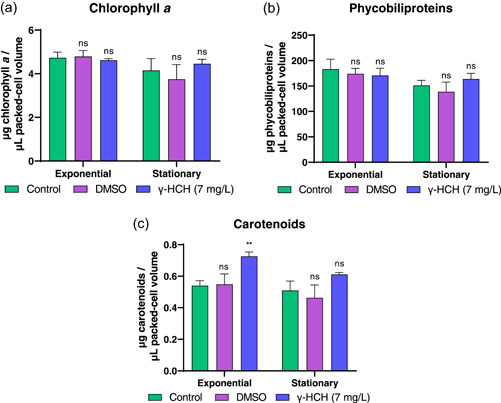
Pigment composition of *Anabaena* sp. PCC 7120 cells treated with 7 mg/L of lindane. The content of chlorophyll *a* (a), phycobiliproteins (b), and carotenoids (c) in *Anabaena* sp. PCC 7120 cells exposed to 7 mg/L of lindane was determined at exponential (OD_750nm_ = 1.0) and stationary (OD_750nm_ = 2.0) growth phases. Since DMSO was used to dissolve lindane in the preparation of stock solutions, a control culture in which the same amount of DMSO alone was added to cells is also included. Three biological replicates were used for each sample. Values were normalized to packed cell volume and expressed as micrograms of pigment × μL**^−^** ^1^ packed cell volume. The average of the three measurements is represented and the standard deviation is included. ns not significant, **p < 0.01, obtained with a *t*‐test analysis comparing each data with respect to its control.

To better understand the physiological responses of *Anabaena* sp. PCC 7120 to lindane, pigment composition (chlorophyll *a*, phycobiliprotein, and carotenoid contents) was determined in both exponential and stationary growth phases. The raw values of pigment content were normalized by using the PCV. As can be seen in Figure 2a,b no changes were observed in the level of chlorophyll *a* and phycobiliproteins present in cells treated with lindane related to those in nontreated cells. However, the level of carotenoids showed a moderated increase in cells treated with lindane in exponential phase, although afterward in stationary phase they remained similar to the untreated cultures (Figure 2c). Taken together, these data indicate that lindane at 7 mg/L concentration barely affects the growth and pigment composition of *Anabaena* sp. PCC 7120.

### 3.2 The presence of lindane altered the photosynthetic electron transport and respiration of *Anabaena* sp. PCC 7120 cells

**Figure 3:**
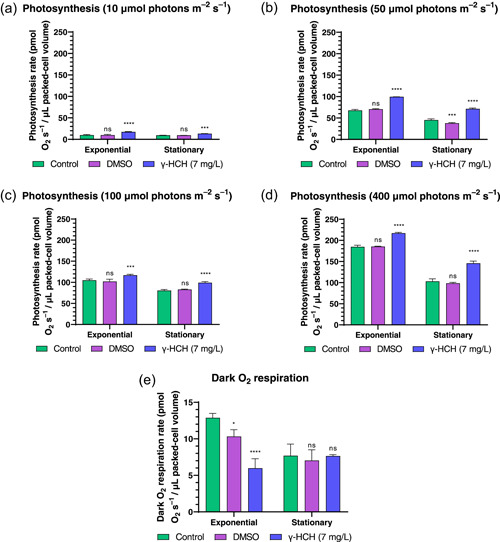
Net photosynthesis and dark respiration rates of *Anabaena* sp. PCC 7120 cells in the presence of 7 mg/L of lindane. Net photosynthesis rate at different light intensities ([a] 10, [b] 50, [c] 100, and [d] 400 μmol photons m^–2^ s^–1^) and dark respiration rate (e) were measured at exponential (OD_750nm_ = 1.0) and stationary (OD_750nm_ = 2.0) growth phases in *Anabaena* sp. PCC 7120 cells exposed to 7 mg/L of lindane. Since DMSO was used to dissolve lindane in the preparation of stock solutions, a control culture in which the same amount of DMSO alone was added to cells is also included. Three biological replicates were used for each sample. Values were normalized to packed cell volume and expressed as pmol O_2_ s**^−^** ^1^ × μL**^−^** ^1^ packed cell volume. The average of the three measurements is represented and the standard deviation is included. ns, no significant, *p < 0.05, ***p < 0.001, ****p < 0.0001, obtained with a *t‐*test analysis comparing each data with respect to its control.

The photosynthetic rate was measured by using different light thresholds (10, 50, 100, 400 μmol photons m^−2^s^−1^) in *Anabaena* cultures grown in the presence and absence of lindane. Results revealed that the presence of lindane increased the photosynthetic rate in *Anabaena* cells in both exponential and stationary growth phases under the four conditions tested (Figure 3a–d). Nevertheless, the respiration rate decreased in the lindane‐treated cells in exponential growth phase whereas no changes were observed in stationary growth phase (Figure 3e).

### 3.3 Lindane triggered oxidative stress in *Anabaena* cells

**Figure 4:**
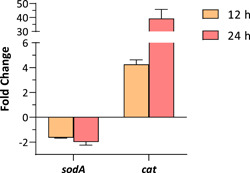
Relative transcription of genes involved in oxidative stress response in *Anabaena* sp. PCC 7120 cells treated with 7 mg/L of lindane. The relative transcription of superoxide dismutase A (*sodA*) and catalase (*cat*) was analyzed by real‐time PCR (RT‐PCR) in *Anabaena* sp. PCC 7120 cells after 12 and 24 h of exposure to 7 mg/L of lindane with respect to untreated cells. Values are expressed as fold change (treated vs. control) and correspond to the average of three biological and three technical replicates. The standard deviation is indicated.

Lindane has been described as a cause of oxidative stress in many organisms, including cyanobacteria (Ceballos‐Laita et al., 2015; Deng et al., 2022; Yu et al., 2020). To study whether lindane can induce oxidative stress response, the expression of two key enzymes involved in the detoxification of reactive oxygen species (ROS) was analyzed in *Anabaena* sp. PCC 7120 culture after 12 and 24 h of treatment with 7 mg/L of lindane. The selected enzymes were superoxide dismutase A (*sodA*) and catalase (*cat*). Results shown in Figure 4 indicated that the expression of *sodA* was slightly downregulated but the expression of cat was strongly upregulated in these conditions (four‐fold after 12 h of lindane treatment and 40‐fold after 24 h of lindane treatment). In addition, superoxide dismutase and catalase activities were also determined in cultures after their exposure to 7 mg/L of lindane. Results are shown in Table 1. Catalytic activities were in agreement with transcriptional data since a significant increase in catalase activity was observed in cells exposed to lindane whereas the SOD activity was not altered in these conditions (Table 1). In summary, these results strongly suggest the induction of the oxidative stress response in *Anabaena* sp. PCC 7120 cells as a consequence of the presence of lindane.

**Table 1:** Enzymatic activities.

Enzymatic activities (U/mg total protein)	*Anabaena* sp. PCC 7120	*Anabaena* sp. PCC 7120 + 7 mg/L γ‐HCH
Superoxide dismutase activity	2.93 ± 0.13	2.74 ± 0.05^ns^
Catalase activity	11.01 ± 1.03	24.4 ± 2.1*

*Note*: Enzymatic activities were subjected to a *t*‐test with respect to the control to determine if values were significant: ns, not significant.

* *p* < 0.05.

### 3.4 *Anabaena* sp. PCC 7120 cells were able to metabolize lindane

**Figure 5:**
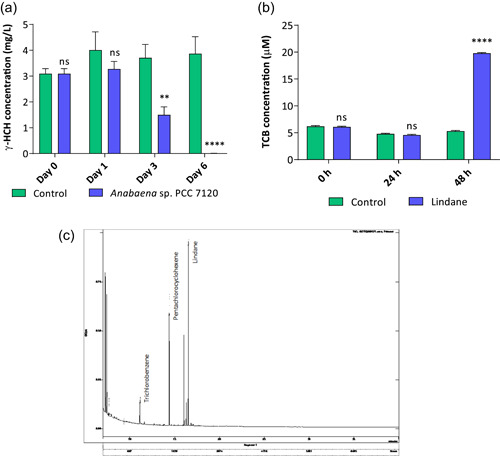
Analysis of lindane degradation by *Anabaena* sp. PCC 7120. (a) Lindane concentration in the supernatant of *Anabaena* sp. PCC 7120 cultures containing 7 mg/L of lindane after 1, 3, and 6 days of exposure. (b) Trichlorobenzene (TCB) concentration inside *Anabaena* sp. PCC 7120 cells after 0, 24, and 48 h of exposure to 7 mg/L of lindane. ns, not significant; **p < 0.01, ****p < 0.0001, obtained with a *t*‐test analysis comparing each data with respect to its control. (c) Chromatogram generated by GCMS analysis of extracts retrieved from *Anabaena* sp. PCC7120 cells treated with 7 mg/L of lindane for 48 h. The products found in the analyses (trichlorobenzene, pentachlorocyclohexene, and lindane) are indicated in the chromatogram.

Lindane degradation experiments were performed with *Anabaena* PCC 7120 by using a BG11‐modified media lacking ammonia to avoid inhibition of lindane metabolization (Kuritz & Wolk, 1995). Cultures were incubated with 7 mg/L of lindane in triplicated and aliquots of the supernatants were taken at different times to determine the decrease in lindane content. Data shown in Figure 5a indicated that *Anabaena* was able to metabolize lindane almost totally after 6 days of the experiment. The degradation seemed to start after 1 day of exposure to lindane and its concentration decreased to 40.43% after 3 days of incubation and 99.75% after 6 days of incubation (Figure 5a).

The presence of some metabolic intermediaries of the lindane degradation pathway of *S. paucimobilis* was analyzed by GC‐MS inside of the *Anabaena* sp. PCC 7120 cells were treated with lindane after 24 and 48 h of treatment. The intracellular intermediates analyzed were pentachlorocyclohexene and trichlorobenzene (resulting products of LinA enzyme), 2‐5‐dichlorophenol (resulting product of LinB enzyme), 2,5‐dichlorohidroquinone (resulting product of LinC enzyme) and chlorohydroquinone (resulting product of LinD enzyme) (Nagata et al., 2007). Trichlorobenzene and pentachlorocyclohexene were detected in the cells after 48 h of lindane treatment (Figure 5b,c). However, only TCB was quantified because pentachlorocyclohexene probably is the product of a first step of dechlorination that in turn is transformed into trichlorobenzene in a second step of dechlorination as happens in the first step of lindane degradation performed by *linA* enzyme in *S. paucimobilis*. The intermediaries 2‐5‐dichlorophenol, 2,5‐dichlorohidroquinone, and chlorohydroquinone were not found inside *Anabaena* sp. PCC 7120 cells.

### 3.5 *Anabaena* genome contains some putative *lin* genes

Orthologs of *lin* genes from *S. paucimobilis* were searched into the genome of *Anabaena* PCC 7120 by performing protein BLAST analyses (Table 2). In the case of *linB*, (haloalkane dehalogenase) two possible orthologs were found. The gene of *Anabaena* sp. PCC 7120 with the highest similarity was *all1353* (41% similarity), which was then called *linB1* (Appendix: Figure A1a). However, another ortholog (*all0193*) was also found. Its similarity, albeit lower, was also significant (33%) but was interestingly annotated as a haloalkane dehalogenase, and was then called *linB2* (Appendix: Figure A1b). These in silico analyses also showed a possible ortholog for the gene *linC* (2,5‐dichloro‐2,5‐ciclohexadiene‐1,4‐diol dehydrogenase), the gene *all3836*, exhibiting a 50% of similarity (Appendix: Figure A1c). Interestingly, a *S. japonicum* UT26 *linC* ortholog was previously described in *M. aeruginosa* NIES‐834 and a phylogenetic analysis of the presence of *linC* in cyanobacteria also found *all3836* as the putative *linC* gene in *Anabaena* sp. PCC 7120 (Sarasa‐Buisán et al., 2022). Protein sequence alignments of LinE (hydroquinone 1,2‐dioxygenase) *and* LinR (transcriptional regulator) of *S. paucimobilis* displayed some degree of homology with the genes products of *all0352* and *alr0353* from *Anabaena* PCC 7120, respectively (26.2% in the case of LinE and 46.7% in the case of LinR) (Appendix: Figure A1d,e). However, the similarity between LinE from *S. paucimobilis* and All0352 from *Anabaena* sp. PCC 7120 is slightly low (26.2%), it is divergently located with respect to *alr0353*, which is in agreement with *linR* and *linE* arrangement in the *Sphingomonas* genome, suggesting that both genes could be orthologs of *linR* and *linE*. Finally, these in silico approaches did not allow the identification of orthologs for *linA* (HCH dehydrochlorinase) and *linD* (2,5‐dichlorohidroquinone dechlorinase) in *Anabaena* sp. PCC 7120 genome.

**Table 2:** Results of the identification of putative *lin* genes in *Anabaena* sp. PCC 7120 genome.

*Sphingomonas paucimobilis* B90A			*Anabaena* sp. PCC 7120			Pairwise alignment		
Gene	Annotation	Protein length	Gene	Annotation	Protein length	Identity	Similarity	Gaps
*linA*	Gamma‐hexachlorocyclohexane dehydrochlorinase	156 aa	_	_		_	_	_
*linB*	Haloalkane dehalogenase	296 aa	*all1353*	Putative hydrolase	282 aa	23.5%	40.6%	6.04%
			*all0193*	Haloalkane dehalogenase	292 aa	17.5%	33.3%	5.94%
*linC*	2,5‐dichloro‐2,5‐cyclohexadiene‐1,4‐diol dehydrogenase	250 aa	*all3836*	Glucose 1‐dehydrogenase	269 aa	30.6%	49.8%	8.5%
*linD*	2,5‐dichlorohydroquinone reductive dechlorinase	346 aa	*_*	_		_	_	_
*linE*	Chlorohydroquinone/hydroquinone 1,2‐dioxygenase	321 aa	*all0352*	NADPH‐dependent carbonyl reductase	248 aa	11.7%	26.2%	24.9%
*linR*	HTH‐type transcriptional regulator LinR	303 aa	*alr0353*	Transcriptional regulator	184 aa	25.5%	46.7%	1.3%

### 3.6 Dynamics of expression of putative *lin* genes in *Anabaena* PCC 7120

**Figure 6:**
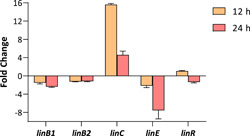
Relative transcription of putative *lin* genes in *Anabaena* sp. PCC 7120 cells treated with 7 mg/L of lindane. The relative transcription of putative *lin* genes (*linB1*, *linB2*, *linC*, *linE*, and *linR*) was analyzed by real‐time PCR (RT‐PCR) in *Anabaena* sp. PCC 7120 cells after 12 and 24 h of exposure to 7 mg/L of lindane with respect to untreated cells. Values are expressed as fold change (treated vs. control) and correspond to the average of three biological and three technical replicates. The standard deviation is indicated.

Given the presence of some putative *lin* genes in the genome of *Anabaena* sp. PCC 7120, the expression of *linB1*, *linB2*, *linC, linE*, and *linR* was analyzed by using Real‐Time RT‐PCR. *Anabaena* sp. PCC 7120 cells were exposed to 7 mg/L of lindane and gene expression was analyzed after 12 and 24 h of treatment. As can be seen in Figure 6, a clear induction of *linC* (16‐fold) was observed after 12 h of treatment. Nevertheless, after 24 h of treatment, the induction was softened to four‐fold. On the contrary, the levels of expression of *linE* decreased after 24 h of exposure. The transcription of the rest of the genes (*linB1*, *linB2*, and *linR*) remained unaltered.

## 4 DISCUSSION

**Figure A2:**
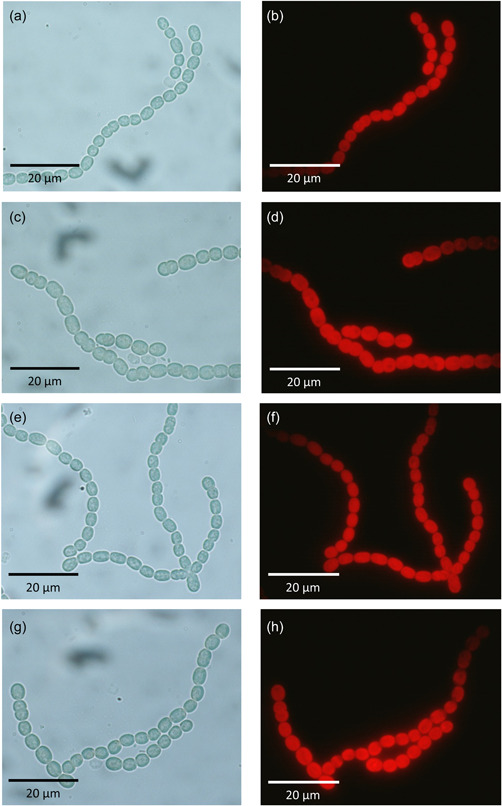
Analysis of the effects of 7 mg/L lindane on cell morphology. Bright‐field microscopic examinations of *Anabaena* sp. PCC 7120 untreated cells at exponential (a) and stationary (e) growth phases and cells in the presence of 7 mg/L of lindane at exponential (c) and stationary (g) growth phases. Fluorescence microscopic examination of *Anabaena* sp. PCC 7120 untreated cells at exponential (b) and stationary (f) growth phases and cells in the presence of 7 mg/L of lindane at exponential (d) and stationary (h) growth phases. Photomicrographs of each condition are representative of at least 10 different images from three biological replicates (scale bars: 20 μm).

*Anabaena* sp. PCC 7120 is a model cyanobacterium that exhibits wide biotechnological applications (Abed et al., 2009). The use of *Anabaena* sp. PCC 7120 in bioremediation processes such as bioaugmentation of lindane‐contaminated waters is conditioned by several factors including its resistance to the presence of this organochlorine pesticide. In the present work, we show data relative to the growth, pigment composition, and photosynthetic/respiration rate of *Anabaena* sp. PCC 7120 in the presence of lindane at its solubility limit in water. Our results indicate that 7 mg/L of lindane barely affects the growth and pigment composition of *Anabaena* sp. PCC 7120, suggesting a suitable physiology state of the cells under these conditions. This fact is in agreement with no morphological differences observed in *Anabaena* sp. PCC 7120 cultures treated with lindane both in exponential and stationary growth phases (Appendix: Figure A2). However, the enrichment of antenna complexes in carotenoids is a well‐established process that allows the dissipation of free radicals generated by oxidative stress in cyanobacteria (Xiao et al., 2011). Thus, although cells seem not to be affected by lindane, the increase in carotenoid content joined to the strong upregulation of catalase expression suggest that lindane is triggering the oxidative stress response in *Anabaena* cells.

Surprisingly, we observed an increase in the photosynthetic rate in *Anabaena* cells treated with lindane. Although these data had been previously reported (Bueno et al., 2004), these results seem to be quite intriguing. One possible explanation could be the fact that as the measurement of photosynthesis is performed determining the release of oxygen, other processes different from photosynthesis could be triggering oxygen production in the presence of lindane, for example, the oxidative stress response via catalase. This hypothesis would be congruent with our observation of upregulation in the expression of catalase and the higher activity of catalase in the cells in the presence of lindane. On the other hand, the data on the decrease in the respiration rate in cells treated with lindane is highly interesting since it might suggest that *Anabaena* cells are not utilizing lindane as a carbon and energy source as happens in *Sphingomonas* cells (Nagata et al., 1999), at least in the conditions tested in the present work. It is important to note that *Anabaena* is a photoautotrophic organism and, under light conditions, this organism uses preferably photosynthetic‐derived carbon sources for growth. However, the degradation of lindane by *Anabaena* grown under dark conditions has never been studied. On the other hand, some bacteria uptake accidentally compounds that they would not metabolize, but if they have enzymes with a low degree of specificity for their main substrates, these compounds can be transformed by these enzymes in other products, and this could be the case of *Anabaena* and lindane. Finally, the reason why the respiration rate decreased in *Anabaena* cells in the presence of lindane is unknown but maybe the alterations in the expression of enzymes that carry out the oxidative stress response in the darkness can be modifying the consumption of oxygen as happened in the production of oxygen by photosynthesis in the presence of lindane.

Regarding lindane metabolization, in this work, we observed that lindane content decreased in supernatants of *Anabaena* sp. PCC 7120 cultures. Simultaneously an increase in the presence of trichlorobenzene is observed inside *Anabaena* sp. PCC 7120 cells. These data are in agreement with previous results reported by Kuritz and Wolk (1995), but the difference relies on the amount of lindane added to the cultures. In this work, 7 mg/L of lindane was added to the cultures, whereas in the experiments performed by Kuritz and Wolk, assays were carried out by using 0.5 μg/L of lindane. In this work, the authors found an increase in γ‐pentachlorocyclohexene and 1,2,4‐trichlorobenzene inside the cells treated with lindane. Since both metabolites disappeared over time, our interest was to find other metabolic intermediaries inside *Anabaena* cells that were similar to those of the *Sphingomonas* lindane degradation pathway. However, this task was unsuccessful because 2‐5‐dichlorophenol (resulting product of LinB enzyme), 2,5‐dichlorohidroquinone (resulting product of LinC enzyme), and chlorohydroquinone (resulting product of LinD enzyme) were not detected inside *Anabaena* cells. Intriguingly, the protein sequence similarity searches of *lin* genes indicated that *Anabaena* contains two putative orthologs of *linB* (*linB1* and *linB2*) and *linC* genes so that the enzymatic reactions catalyzed by these enzymes, as well as the degradation pathway after TCB remains unknown. According to our search of putative *lin* genes, an ortholog of *linA* (HCH dechlorinase) was not found in the genome of *Anabaena* sp. PCC 7120. In *Sphingomonas* two initial dechlorination reactions carried out by *linA* produce 1,3,4,6‐tetrachloro‐1,4‐cyclohexadiene which presumptively is spontaneously dechlorinated to yield 1,2,4 trichlorobenzene. Since we found 1,2,4 trichlorobenzene inside *Anabaena* cells, another HCH dechlorinase or a similar, functionally equivalent enzyme must be catalyzing this reaction in this cyanobacterium.

In relation to *Sphingomonas*, although *lin* genes in *Anabaena* are just proposed, it is interesting to note the features concerning *lin* gene expression. In *Sphingomonas*, *linB* and *linC* are constitutively expressed whereas in *Anabaena*, the potential *linB* genes are constitutively expressed but the expression of the proposed *linC* gene is clearly inducible in the presence of lindane. Interestingly this *linC* induction was also observed in *M. aeruginosa* PCC 7806 cells (Sarasa‐Buisán et al., 2022). The presence of *lin* genes and the inexistence of some metabolic intermediaries seem to suggest some alternative lindane metabolization pathway in *Anabaena* although an uncompleted pathway cannot be ruled out in which trichlorobenzene would be a dead‐end product. Consequently, carefully designed experiments should be carried out in the future to clarify the lindane degradation pathway of *Anabaena* sp. PCC 7120.

In conclusion, the information provided in this work contributes to a better understanding of the physiological and transcriptional responses of *Anabaena* sp. PCC 7120 to lindane which would be helpful in the utilization of this cyanobacterium alone or taking part of consortia in the bioremediation of this pesticide. *Anabaena* sp. PCC7120 could be used in freshwater systems as the first step of lindane bioremediation to generate trichlorobenzene. Then, trichlorobenzenes can be reductively dechlorinated by organohalide‐respiring bacteria in genera *Dehalococcoides* (Hölscher et al., 2003), *Dehalobacter* (Nelson et al., 2011), *Dehalobium* (Wu et al., 2002) and *Dehalogenimonas* (Qiao et al., 2018) or could be aerobically degraded by Proteobacteria such as *Pseudomonas*, *Sphingomonas*, and *Xantobacter* (Field & Sierra‐Alvarez, 2007). Finally, it is interesting to point out that in this work, we also found a gene that is remarkably induced in the presence of lindane in *Anabaena* sp. PCC7120. This result is of great interest because this induction can be used in the development of sensing circuits for the generation of whole‐cell biosensors based in the host *Anabaena* sp. PCC7120 for the detection of lindane.

## AUTHOR CONTRIBUTIONS

**Jorge Guío**: Data curation (equal); formal analysis (equal); investigation (equal); methodology (equal). **Maria F. Fillat**: Funding acquisition (equal); supervision (equal). **Maria L. Peleato**: Conceptualization (equal); funding acquisition (equal); supervision (equal). **Emma Sevilla**: Conceptualization (equal); formal analysis (equal); supervision (equal); writing—original draft (lead); writing—review and editing (lead).

## CONFLICT OF INTEREST STATEMENT

None declared.

## ETHICS STATEMENT

None required.

## Data Availability

All data are provided in full in the results section of this paper and its appendix.

## 1

**Table A1:** Oligonucleotides used in this study.

Primer	Sequence (5′–3′)	Gene
*rnpB* for	AGCGGAACTGGTAAAAGACCAA	Gene *rnpB* from *Anabaena*
*rnpB* rev	GAGAGGTACTGGCTCGGTAAACC	sp. PCC 7120
*linB1* for	CAGCTTTGGCGGCTCAAG	Gene *all1353* from *Anabaena*
*linB1* rev	GAAAAGCCAGAACCAATCCAAT	sp. PCC 7120
*linB2* for	CGATCGCACTCTCAAAGCTATAATC	Gene *all0193* from *Anabaena*
*linB2* rev	TCACATAGTAGCGCCAGATATGC	sp. PCC 7120
*linC* for	GGAAAAGAAAGCAGTTGTAGAAAGTCA	Gene *all3836* from *Anabaena*
*linC* rev	GCTACTGCTGCCGCCATTT	sp. PCC 7120
*linE* for	ACTTCCGCATCTCTGCAAAAA	Gene *all0352* from *Anabaena*
*linE* rev	CAGCCGTCTATTTGGCAACA	sp. PCC 7120
*linR* for	AAATTCATGTTGCGTTGGGTTT	Gene *alr0353* from
*linR* rev	CCAACAAAGTGTTCTTCAAATAATGG	*Anabaena* sp. PCC 7120
*sodA* for	CTAACCAAACCCAACCACTACCA	Gene *sodA* from *Anabaena*
*sodA* rev	CCTTTGGCAGTTTTGAAGAGTTC	sp. PCC 7120
*catA* for	ACCCACGGGATGTCATTATGA	Gene *catA* from *Anabaena*
*catA* rev	TGGCAACGCACCTAAACCA	sp. PCC 7120
